# Neurologic complications after cardiopulmonary bypass – A narrative review

**DOI:** 10.1177/02676591221119312

**Published:** 2022-08-19

**Authors:** Tom Gilbey, Benjamin Milne, Filip de Somer, Gudrun Kunst

**Affiliations:** 1 Department of Anaesthesia & Pain Medicine, 8948 King’s College Hospital NHS Foundation Trust, London, UK; 2 Department of Human Structure and Repair, Faculty of Medicine and Health Sciences, 26656 Ghent University Hospital, Ghent, Belgium; 3 School of Cardiovascular Medicine and Sciences, Faculty of Life Sciences and Medicine, 111990 King’s College London British Heart Foundation Centre of Excellence, London, UK

**Keywords:** Cardiac surgery, postoperative neurological complications, postoperative stroke, postoperative delirium, postoperative neurocognitive disorders, postoperative cognitive dysfunction

## Abstract

Neurologic complications, associated with cardiac surgery and cardiopulmonary bypass (CPB) in adults, are common and can be devastating in some cases. This comprehensive review will not only consider the broad categories of stroke and neurocognitive dysfunction, but it also summarises other neurological complications associated with CPB, and it provides an update about risks, prevention and treatment. Where appropriate, we consider the impact of off-pump techniques upon our understanding of the contribution of CPB to adverse outcomes.

## Introduction

The classification of neurological complications after adult cardiac surgery depends upon the background and perspective of the clinician, and the tools at their disposal. Surgical authors have focussed on the resolution of symptoms leading to the classification of temporary or permanent neurological deficits; this has been particularly useful in the literature relating to deep hypothermic cardiac arrest (DHCA). By contrast, neurologists are often consulted to define a particular diagnosis, a process which may consider the symptoms and pathophysiology of the deficit more in detail. As multidisciplinary perioperative care of cardiac surgical patients has evolved, the distinction between temporary and permanent has become less clear. This is due to more advanced cognitive assessments, longer follow ups, and new imaging tools. Separation into distinct diagnoses may facilitate strategies to mitigate operative risk, even if the underlying causes include a continuum of disease related to patient-specific vascular risk factors. Advances in epidemiology have enabled us to view the perioperative period against a broader context of long-term vascular outcomes without surgery.

This comprehensive narrative review article provides an update about the broad categories of stroke, neurocognitive dysfunction and other neurological diagnoses such as seizures or visual loss. Throughout the manuscript we will consider the impact of off-pump techniques with the view to further understand the impact of cardiopulmonary bypass (CPB) to neurological complications. Table 1 provides a list of possible neurological complications after cardiac surgery, selected risk factors, incidence and onset.^1–10^

**Table 1.** Neurological complications of cardiopulmonary bypass.

Complication	Time	Incidence	Risk factors	Key references
Stroke	Early	1–17%	Atherosclerosis, valve, DHCA	Messe et al. 2014^1^
	Late	1%	Atrial fibrillation	Gaudino et al. 2019^2^
Seizures	Immediate	1–8%	Tranexamic acid	Pataraia et al. 2018^3^
Delirium	Immediate	1–50%	Vascular risk, age, emergency (Table 2)	Rudolph et al. 2009^5^
Coma	Immediate	2.8%	AKI, anaemia, emergency (Table 3)	Rodriguez et al. 2011^4^
Cognitive disturbance	Long term	1–42%	Controversial; vascular risk, frailty	McKhann et al. 2005^6^
Visual loss	Long term	0.1%	Vascular risk	Kalyani et al. 1987^7^
Saccadic abnormality	Unclear	Unclear	Aortic valve surgery	Solomon et al. 2008^8^
Phrenic nerve palsy	Unclear	26%	Pericardiac ice slush	DeVita et al. 1993^9^
Chorea	Long term	Rare	DHCA, complexity	Medlock et al. 1993^10^

DHCA: deep hypothermic cardiac arrest.

## Stroke

Stroke is a feared outcome of cardiac surgery. During the early years of the coronary artery bypass grafting (CABG) procedure, primary cardiac death was the most common complication. As technical improvements reduced this as a source of mortality, morbidity and dependence, the epidemiology of stroke came more and more into focus.^11,12^

Patients suffering perioperative stroke have worse clinical outcomes beyond the associated disability with the condition. Dacey et al. performed a prospective cohort study of 575 patients who had CABG alone.^13^ The overall incidence of stroke was 1.6% and there was a threefold increased risk of death during 10 years after surgery in patients with stroke. Stroke-induced poorer clinical outcomes have been shown to be associated with increased hospital costs.^14^

Across all cardiac surgery, CPB was assumed to be the key causative mechanism of stroke, however, the advent of off pump CABG surgery (OPCAB) has allowed the direct comparison of stroke risk with and without bypass and no significant difference has been found.^15^

### Pathophysiology of postoperative stroke

Stroke associated with cardiac surgery can be divided into early and late, based upon the presence of the deficit at the point of extubation. Most authors have considered the former to be predominantly related to arterial emboli from aortic manipulation, and the latter related to postoperative atrial fibrillation, cerebrovascular disease or potentially associated with haemodynamic instability. The common types of emboli are thromboemboli, atheroemboli, fat emboli and air emboli. Transcranial Doppler (TCD) ultrasonography can discriminate between solid and gas.^16^ It has been suggested that gaseous emboli are more frequent but less harmful than solid. Fat emboli are distributed according to their state of emulsification.^17^ They are deformable and therefore thought to be the most difficult to remove, although the use of a cell saver may be effective.^18^ Regardless of type, all emboli are undesirable. The frequency of emboli detected by TCD is markedly higher during placement of the aortic cannula as well as clamping and unclamping of the aorta.^19^

One method that has been employed to reduce the impact of emboli is the use of temporary intra-arterial filters. These are deployed distal to the cross-clamp to protect the supra-aortic vessels, with the hope that other vulnerable visceral tissues such as the kidneys will also benefit. In a large randomised study, Banbury et al. showed that one device caught emboli in 97% of cases.^20^ However, this study and further attempts in the intervening years have not provided prospective evidence of a reduction in clinical stroke, probably because the studies have been underpowered to detect a difference in this endpoint. Some commentators are persuaded by the high rate of embolus capture but others feel that such devices increase operative time and cost, often require another small aortic cannulation, and may disrupt but not catch atheroma at the origins of the arch vessels. It seems unlikely that this question will be definitively answered for cardiopulmonary bypass cases but there is great interest in filters for transcatheter aortic valve implantation (TAVI).

Cerebral hypoperfusion is an important cause of intraoperative stroke. Systemic vasodilatation and reduced cardiac output when not on CPB may result in hypotension. Intraluminal stenosis due to atherosclerotic disease and the reduced flow to vulnerable “watershed” regions at the boundaries between major cerebral artery territories further increase the risk. Stroke mechanisms do not operate in isolation and low flow regions will be more likely to collect microemboli. It has been shown that unilateral watershed strokes are caused by emboli, whereas bilateral are due to systemic hypotension.^21^

Primary haemorrhagic stroke related to cardiac surgery is rare but approximately 5% of perioperative ischaemic strokes will have some degree of haemorrhagic transformation (HT).^22^ The incidence of HT in the general ischaemic stroke literature varies greatly depending upon whether the diagnosis is made on imaging or via post-mortem, and depending upon the specific radiological criteria used.^23^ This usually does not have a significant effect on the size, but large infarcts and anticoagulation increase the risk. Those patients requiring valve replacements due to infective endocarditis may be at greater risk of haemorrhagic stroke due to ongoing infective embolization.^24^ One strategy to manage this would be to give antibiotics and delay operating until the risk is reduced, provided that the patient is not haemodynamically unstable.

Clearly these are complex and individualised decisions requiring substantial multidisciplinary input. Some authors argue that earlier surgery can produce more favourable outcomes with modern techniques and neuroimaging to guide decision-making. This is especially true with virulent infectious organisms and rapid progression of cardiac failure. There is very little high-quality evidence to guide treatment. However the American Heart Association (AHA), European Society of Cardiology (ESC) and Society of Thoracic Surgeons (STS) all produce infective endocarditis guidelines which agree on a delay of at least 4 weeks for surgery after haemorrhagic stroke, and the AHA and STS are similarly conservative after significant ischaemic strokes. The rationale for early surgery and guideline content is elegantly summarised by Yanagawa and colleagues.^25^

A meta-analysis of trials consisting of all procedures showed an even split between early and late strokes with a pooled event rate of just below 1% for each category.^2^ In an earlier study of CABG surgery, 40% of strokes were intraoperative, and postoperative strokes peaked at 40 h^26^ The timepoint of the postoperative stroke may reflect a situation where anticoagulation is not desirable but cardioembolic risk is already present. Interestingly, postoperative stroke was not associated with new-onset postoperative AF in the latter study.^26^

The systemic inflammatory response provoked by cardiac surgery and CPB may be more important. This is thought to peak on the first two perioperative days and in older adults undergoing non-cardiac surgery haematological indices of inflammation are correlated with stroke risk.^27,28^ It is important to note that intraoperative strokes are unlikely to recur, but a postoperative stroke may suggest ongoing risk, whether from inflammation and endothelial dysfunction, arrythmias or residual intracardiac thrombus. The largest studies have shown no difference in overall stroke rates in patients undergoing CABG with or without CPB, suggesting that the use of CPB does not contribute to the risk of strokes.^29^

### Procedural risk for stroke

It is difficult to standardise risk profiles for cardiac surgery between surgeons and across institutions because of variations in technique. The Society of Thoracic Surgeons risk calculator (STS, https://riskcalc.sts.org/stswebriskcalc/calculate) gives an assessment of the risk of stroke (defined as an acute episode of focal or global neurologic dysfunction for longer than 24 h) as a specific outcome. The risk is much higher after multiple procedures (such as CABG plus valve) with 2.5%, when compared to single cardiac surgical procedures.^30^ This is based upon North American data and performs well for a restricted subset of procedures.

In addition, valve procedures are generally agreed to have a higher risk of stroke than CABG alone. This reflects the fact that cardiac chambers have been opened. In a prospective multicentre cohort study by Wolman et al. including 273 patients, the risk for severe adverse neurological outcomes (including stroke) was 8.4% for combined valve and CABG procedures against 3.1% for CABG alone.^31^ Another two-centre prospective study in 196 patients, 65 years or older undergoing aortic valve replacement for aortic stenosis, optimised the protocol to be highly sensitive for stroke by including clinical assessments by neurologists before and after the procedure. They showed that 17% of patients had clinical strokes and 54% of the remainder had new lesions on magnetic resonance imaging (MRI).^1^ This is substantially higher than that predicted for the same procedure by the STS data, which was 7% at that time. Diffusion-weighted MRI imaging is the gold standard modality for detecting novel postoperative acute cerebral ischaemic events and a meta-analysis showed an overall incidence of silent new MRI diagnosed brain lesions of 29% after all types of cardiac surgery.^32^

These MRI sequences may be impractical to perform on clinically unstable patients but may show areas of cerebral ischaemia within minutes of the infarct occurring. Non-contrast computed tomography (CT) scans may show only very subtle changes within 24 h of onset but may be helpful to exclude haemorrhage.^33^ Advanced imaging such as CT perfusion scans can help to define the potential benefits of thrombectomy in embolic stroke, which may be the only viable treatment option in a patient who has recently had cardiac surgery and is thus at high bleeding risk.

The risk of stroke for procedures that require DHCA is thought to be among the highest of all. Ergin and colleagues found that 11% of patients in a retrospective cohort had strokes with more than half of these patients suffering permanent deficits.^34^ These were predominantly embolic in nature and the risk was increased when operating on aneurysms containing clot or atheroma. It is difficult to separate the risks related to the pathology treated from those related to the perfusion technique and there is substantial mortality in this patient group. More recently, some centres report much lower rates of 2.1% for elective cases under DHCA.^35^ It is not clear if this variability is related to case mix, surgical experience and technique or refinement over many years of experience.

### Patient-related risks for stroke

A very broad range of risk factors have been associated with stroke. Perhaps the strongest of these is a history of prior stroke, with an odds ratio of approximately four.^36^ Both transient ischaemic attacks and silent cerebral ischaemia, as defined by asymptomatic ischaemic brain lesions found on MRI, are similarly associated.^37^ Increasing age, female sex, diabetes, peripheral arterial disease, renal failure and hypertension have all been reported as increasing the risk of stroke.^11,14,38^

Females have been reported to be at 3-fold higher risk of perioperative stroke in a large series of 2972 cardiac surgery patients by Hogue and colleagues after adjusting for confounders.^39^ Females have been shown to have a greater burden of cerebral atherosclerosis beyond the age of 65, leading investigators to hypothesise regarding the protective role of oestrogen in this process.^40^ However, recent work has shown that postmenopausal time is not an independent risk factor for asymptomatic intracranial atherosclerosis.^41^ Therefore, the interaction of female sex with other vascular risk factors may not be linear and retrospective studies of perioperative stroke may be subject to residual confounding.

In addition, cardiac diagnoses including low cardiac output syndrome, atrial fibrillation, a recent history of myocardial infarction, and left ventricular dysfunction are also associated with stroke.

### Prevention of stroke or emboli

Patients with significant cardiovascular risk factors should be considered for a statin, close blood pressure control and antiplatelets as appropriate. Preoperatively, especially in high-risk patients, the proximal carotid arteries and the aorta itself are evaluated for atherosclerosis. Stenoses at other sites, particularly the distal internal carotid arteries and the circle of Willis can be common causes of stroke and aggressive medical management of risk factors is the treatment of choice.

The use of carotid endarterectomy (CEA) to reduce the risk of stroke is controversial. Clinicians need to decide if atherosclerosis at the carotid site is dangerous in itself or whether it is a marker for aortic atherosclerosis, which will cause intraoperative stroke when the aorta is cannulated for CPB, or both. Naylor and Bown propose that unilateral, asymptomatic disease should not be treated preoperatively, bilateral severe asymptomatic disease could be treated prophylactically although more data is required to support this recommendation, and that symptomatic disease should always be treated.^42^ CEA has been performed before or during CPB with acceptable outcomes with respect to postoperative bleeding and stroke.^43^

There is consensus that management of an atherosclerotic aorta is central to the prevention of stroke during CPB. Positioning of the cross clamp and the site of cannulation must be carefully chosen. The preoperative uncovering of a porcelain or heavily calcified aorta may be a challenge, but it is important as porcelain aortas cannot be clamped. Intraoperative epi-aortic ultrasound has emerged as a tool to localise plaques and to guide cannulation. Biancari et al. used propensity-matched registry data from CABG patients to show that epi-aortic ultrasound was associated with a reduced stroke risk from 2.6 to 0.6%.^44^ They argued that this technique was superior to palpation or transoesophageal echo and would allow complete avoidance of aortic manipulation in some cases according to surgical judgement. Recent work has considered the concept of an-aortic off-pump CABG (anOPCAB, often described as no-touch techniques).^45^ Historically, partial clamping with aortic side-clamps, manipulation or stabilisation of the aorta has been involved in OPCAB. The various anOPCAB techniques remove this, either with clamp-free proximal anastomoses or via the use of complex branched total arterial revascularisation. In a network meta-analysis including 13 studies with 37,720 patients, Zhao et al. found that anOPCAB reduced the risk of stroke by 78% versus traditional on-pump CABG.^46^ In addition, a range of other outcomes such as 30-day mortality and renal failure were dramatically improved by anOPCAB (i.e., no aortic side-clamps used).

The single largest prospective trial comparing traditional on-pump CABG versus OPCAB (both techniques using side-clamps) is the CORONARY study (4752 patients from 19 countries). This showed no significant differences in postoperative neurological and other clinically relevant organ failure outcomes.^29^ A large meta-analysis of 18,076 CABG patients did show a significant reduction in stroke with OPCAB but this conclusion was weaker when only larger studies with less risk of bias were included.^47^ The simplest way to reconcile the available data is to conclude that CPB itself is not a strong causative factor for stroke but that any surgical manipulation of the aorta including cannulation for bypass may be. It is unlikely that larger trials than CORONARY will be feasible. The benefits of anOPCAB may be confounded by other surgeon-specific factors such as experience and the number of such procedures performed likely remains too small to prove that this is the “gold standard” in stroke prevention using a prospective, randomised study design.

Extreme haemodilution to minimise transfusion has been associated with increased rates of stroke;^48^ this is now avoided, and the American Society of Thoracic Surgeons guidelines and recent European CPB guidelines recommend a minimum haemoglobin of 6 g/dl whilst on bypass.^39,49,50^ Other techniques to optimise and maintain cerebral oxygen delivery during CPB include targeting a minimum oxygen delivery of 273 mL/min/m^2^, which has been recommended by the recent European CPB guidelines.^50^ Furthermore, the use of near-infrared spectroscopy (NIRS) guided algorithms to improve cerebral oxygen delivery has been recommended. However, the evidence is conflicting, and the guidelines suggest only a class IIb recommendation for NIRS, i.e., that this technique may be considered.^50^ A recent narrative review about cerebral monitoring in cardiac surgery discusses the evidence from clinical studies regarding the ability of NIRS to prevent stroke or neurocognitive dysfunction.^51^ Furthermore, multimodal cerebral monitoring is discussed in this comprehensive review, as a possible approach to improve neurological outcomes.^51^

Blood pressure management during CPB is contentious, with conflicting studies that have found increased rates of stroke at both low and high mean arterial pressure (MAP) targets. A recent randomised controlled trial compared a high target of 70–80 mmHg with a low target of 40–50 mmHg in 197 patients and found no significant differences in terms of new ischaemic lesions on MRI.^52^ Variability in blood pressure is associated with increased mortality and cerebral autoregulation is variable between patients.^53,54^ A pragmatic approach is to maintain the MAP at a stable level between 50–80 mmHg, adjusted according to the patient’s baseline blood pressure.^50^ However, in order to resolve the disagreement between studies and to continually improve patient outcomes, a more individualised approach may be needed. Hori et al. used the correlation between TCD-measured blood velocity in the middle cerebral artery and MAP to identify the lower limit of cerebral autoregulation for individual patients.^54^ Worryingly, for 17% of patients a MAP of 78 mmHg was below the lower limit of cerebral autoregulation and magnitude and duration of hypotension below this value was associated with an increased incidence of postoperative stroke. Indeed, a prior study by the same group showed that patients with impaired intraoperative cerebral autoregulation were 4.7-fold more likely to sustain a perioperative stroke than those with intact autoregulation.^55^ Unfortunately, TCD is technically challenging and the autoregulatory range may change during the surgical procedure, particularly during cooling of the patient, which would necessitate repeated measurements. To combat this, similar approaches have been developed using NIRS.^56^

Recent advances in robotics and signal processing have enabled a new generation of devices that can acquire bilateral middle cerebral artery (MCA) doppler signals and maintain this non-invasive monitoring for the duration of the surgery.^57^ They are relatively large but allow the concurrent use of processed EEG and NIRS as well as scalp cooling devices. The continuous data acquisition allows MCA flow to be maintained at baseline even during selective antegrade and retrograde cerebral perfusion. This may give reassurance beyond that afforded by a stable NIRS signal that neuroprotection is adequate, particularly during the long periods of systemic circulatory arrest necessary for complex aortic arch procedures. If there is uncertainty about the patency of the Circle of Willis continuous TCD could reduce the number of selective perfusion cannulae and the pump flows required.

The optimal temperature during CPB for stroke prevention is unknown. Moderate hypothermia (28°–32°C) does not increase the rate of cerebral emboli measured by TCD and does not increase the rate of stroke.^58,59^ Similarly, the effect upon stroke risk of intraoperative hyperglycaemia is not known but hypoglycaemia associated with tight control is harmful; most guidelines therefore recommend keeping blood glucose below 180 mg/dl or approximately 10 mmol/l.^50^

For procedures requiring DHCA, strategies employed for cerebral protection tend to be variable with very little evidence in the literature. The full range of neurological complications, not simply focal stroke, is associated with a duration of circulatory arrest beyond 40 min^60^ Some centres have argued that because many strokes in this patient cohort are embolic, they are related to the surgery and not specifically to the perfusion technique.^35^ In this view, hypothermia is the most important protective factor and even the placement of selective antegrade cerebral perfusion cannulae may worsen the situation by creating more emboli. An opposite view is that meticulous selective cannulation and perfusion enables the use of moderate, rather than deep hypothermia for all but the most complex surgeries. A popular trend in perfusion technique for the repair of acute type A aortic dissections is to cannulate via a graft anastomosed to the right axillary artery. This can be used both to avoid the complications of femoral cannulation and to deliver antegrade cerebral perfusion during the period of circulatory arrest with minimal additional manipulation of the vessels.^61^

The optimal temperature for DHCA has not been established but in one retrospective study cooling to below 20°C increased the rate of persistent stroke from 5 to 12%.^62^ At these low temperatures the majority of cerebral oxygen consumption is provided by soluble oxygen rather than that bound to haemoglobin. Thus, whilst hyper-oxygenation is generally avoided to mitigate free-radical induced damage whilst on bypass the patient requiring DHCA may benefit from hyperoxia, although this evidence was generated in infants who may have very different cerebral vasculature when compared to the adult patient.^63^ Substantial cooling also raises the issue of acid-base management. A pH-stat approach involves the addition of carbon dioxide to the bypass circuit to correct the alkaline shift observed during cooling. This is thought to act as a cerebral vasodilator, which increases cerebral blood flow and therefore oxygen delivery when compared to an alpha-stat approach. Other potential benefits of the added carbon dioxide that have been suggested, include shifting the oxyhaemoglobin dissociation curve to improve oxygen unloading, more uniform cooling, and further suppression of cerebral metabolism. The latter effect has been confirmed by multiple methods in humans using physiologically relevant concentrations of carbon dioxide by Xu and colleagues.^64^ Critics of the pH-stat approach have pointed out that the only prospective evidence of improved outcome is in animal models or infant humans,^65^ whilst the majority of strokes in adult aortic surgery are embolic rather than ischaemic, as discussed above. This would appear to favour the maintenance of coupling between cerebral blood flow and metabolism with alpha-stat management. In adult DHCA some authors have advocated a two-phase approach with pH-stat during cooling when there is most risk of a mismatch between oxygen delivery and demand. Alpha-stat is then used during rewarming to reduce the risk of cerebral oedema.^60^ Finally, early approaches to DHCA often used haemodilution in an attempt to improve microcirculatory blood flow. However, studies of the cerebral microcirculation in piglets undergoing DHCA showed that a haematocrit of 30% was preferable to 10% in this model.^66^ A randomised trial in infants comparing haematocrit targets of 20% versus 30% was stopped early due to poor developmental outcomes at 1 year in the 20% group.^67^ Nevertheless, a 20% lower limit is still common in adult cases.^66^

In the absence of residual intracardiac thrombus, the risk of postoperative stroke should be stratified using familiar tools. For example, a CHADS (congestive heart failure, hypertension, age >75 years, diabetes mellitus, and prior stroke or transient ischemic attack) score of 2 or higher was associated with an increased risk of stroke or death in one study.^68^

A meta-analysis suggested that surgical closure of the left atrial appendage was associated with a reduction in stroke and with all-cause mortality, however there were only two randomised controlled trials available.^69^ Subsequently the recent results of LAAOS III, a multicentre randomised trial of 4770 patients showed a significant reduction in stroke or systemic embolism with similar mortality.^70^ As the intervention is relatively quick and cheap to perform this is likely to be a practice-changing result although without further trials it is not clear if anticoagulation can be discontinued.

### Treatment of postoperative stroke

Stroke is diagnosed by taking a robust clinical history of the type of symptoms and the time course of their onset. This is supported by a neurological examination and finally by brain imaging as discussed above. The diagnosis can sometimes be subtle, even for the expert stroke physician and not all lesions on brain imaging may be accompanied by symptoms. Nussmeier and colleagues found that a postoperative worsening of the National Institutes of Health Stroke Scale was strongly associated with a composite outcome including stroke in 4707 cardiac surgical patients after CPB, thus validating the common NIHSS tool in this setting.^71^ In general, between 4% and 15% of all strokes occur in hospitalised patients and there is ample evidence that management by specialists improves functional outcomes.^72^ However, for cardiac patients with confirmed stroke, treatment can potentially increase bleeding risk and a multidisciplinary perspective will be required to make these decisions. If the patient no longer needs significant cardiovascular or renal organ support, they should be transferred to a specialist stroke ward with support from therapy professionals.

Physiological targets after stroke include maintenance of an appropriate blood pressure and avoidance of hypoxia, fever and hyperglycaemia. Antiplatelet and statin therapy is safe and appropriate for both CABG and stroke patients. Specific acute therapy is more challenging because systemic thrombolysis is contraindicated after cardiac surgery. There is likely to be significant emphasis on endovascular stroke therapy in appropriate patients. However, despite a strong evidence base in the general stroke literature, this requires equipment and expertise and may not yet be available in many settings.

## Neurocognitive disorders

The term ‘perioperative neurocognitive disorders’ has been introduced as an overarching term for cognitive impairment identified in the preoperative or postoperative period including an acute postoperative event (postoperative delirium) and cognitive decline diagnosed up to 30 days after the procedure (delayed neurocognitive recovery) and up to 12 months (postoperative neurocognitive disorder).^73^ Here, we will stick with the terms of postoperative delirium (POD) for an acute postoperative event and postoperative cognitive decline (POCD) for a delayed neurocognitive event up to 12 months postoperatively, as this terminology has been predominantly used in clinical studies.

POD is common after cardiac surgery and the full extent of the clinical picture of delirium may be underappreciated. It is defined by the Diagnostic and Statistical Manual of Mental Disorders (DSM V) as a fluctuating disturbance in attention and awareness that represents an acute change from baseline early after surgery, is accompanied by disturbed cognition or perception, and usually not due to a pre-existing neurocognitive disorder or occurring in context of a severely reduced arousal levels.^74^ Common risk factors are detailed in Table 2.^75^

**Table 2.** Risk factors for postoperative delirium.

Predisposing	Precipitating
Reduced cognitive reserve	Medications or medication withdrawal
Dementia	Anticholinergics
Depression	Muscle relaxants
Advanced age	Antihistamines
Reduced physical reserve	Antispasmodics
Atherosclerotic disease	Opioids
Renal impairment	Antiarrhythmics
Pulmonary disease	Corticosteroids
Advanced age	Polypharmacy
Preoperative beta blockade	Pain
Visual or hearing impairment	Hypoxemia
Alcohol abuse	Electrolyte abnormalities
Malnutrition	Malnutrition
Dehydration	Dehydration
Apolipoprotein E4 genotype	Environmental change (e.g., hospital or ICU admission)
	Circadian rhythm disruption
	Urinary catheter use
	Restraint use
	Infection
	Psychotropic medications
	Antidepressants
	Antiepileptics
	Antipsychotics
	Benzodiazepines

The incidence depends upon the tool used to screen for delirium and therefore varies across the available literature; it should be assessed at up to 1 week after the procedure or until hospital discharge, whichever occurs sooner.^76^ By contrast POCD implies a longer-term deficit and is a frequently discussed topic in cardiac surgery, particularly in patients with frailty, medical comorbidities and pre-existing cognitive impairment. It is often a research outcome, rather than a clinical entity, and is usually diagnosed from 30 days after surgery.^73^

Due to shared risk factors, most authors consider the entities of POD and POCD to overlap and representing similar processes reported at different postoperative stages. In one prospective study, patients with POD were more likely than those without to experience POCD when subjected to neuropsychiatric testing at 6 months.^77^ Delirium is distressing for patients and costly for healthcare systems and further evidence of the long-term negative impact is provided by one study, which showed an association with late mortality, particularly if delirium occurs in patients under the age of 65.^78^ However, any discussion of the risk attributable to CPB must start with the background risk conferred by a long surgery. Despite this, colloquial terms are common to describe a state of neurocognitive disturbance after bypass – ‘pump-head’ or ‘post-perfusion syndrome’.

### Pathophysiology and risk of neurocognitive disorders

There is a wide range of reported incidences of delirium – the upper end of which may be greater than 50%, as a proportion of patients exhibit a hypoactive phenotype which may be more difficult to recognise and diagnose.^5^ Pre-existing neurological disorders such as stroke and dementia, heavy alcohol consumption, increasing age and known cerebral arterial disease are all independent predictors and are common in cardiac surgery patients.^5,14^

POCD is more common than frank delirium and it can occur in up to 79% of patients following CABG.^14,38,79^ Risk factors identified are very similar to those for stroke, and therefore it has been assumed that a vascular mechanism, probably involving microemboli to the cerebral circulation, has a causative role. However, efforts to correlate microemboli with the extent of neurocognitive dysfunction have been inconsistent.^80^ Intraoperative hypotension, particularly in those who are normally hypertensive, and significant oxygen desaturations have been implicated.^81^ As with the literature on stroke, investigators have focussed upon individualising treatment by assessing cerebral autoregulation, either by transcranial doppler or by ultrasound-tagged NIRS monitors. A randomised single centre trial, including 199 patients, older than 55 years undergoing non-emergency cardiac surgery, suggested that assessing the lower limit of cerebral autoregulation before bypass and then maintaining the MAP above this level during bypass reduced delirium significantly from 53% to 38%.^82^

Continuously monitoring oxygen delivery and maintaining it above a critical value is emerging as a key organ protective strategy during bypass.^50^ Leenders et al. found in a retrospective study, including 357 patients undergoing CABG surgery, that patients who developed delirium had significantly lower nadir oxygen delivery values during CPB.^83^ Other factors that are thought to cause or exacerbate cognitive dysfunction include systemic inflammation, general anaesthetic agents, such as benzodiazepines, fever or sepsis in the perioperative period. There is strong consensus that cerebral hyperthermia will increase the metabolic demands placed upon the brain and exacerbate neuronal injury. This will worsen neurological outcomes, whatever the overarching pathophysiological mechanism. For this reason, guidelines recommend limiting the arterial outlet temperature of the bypass machine to 37°C and to pay careful attention to measuring the patient temperature at a site that accurately reflects the temperature of the cerebral blood flow during CPB, such as the nasal temperature.^84^ Slow rewarming is also recommended to avoid the use of excessive temperatures and to minimise the risk of overshoot. This may be exacerbated by the use of bladder and rectal probes that underestimate the brain temperature during rewarming. Clearly, there is a trade-off between the speed of rewarming and the overall duration of bypass and the procedure itself.

When prospective studies have looked at neurocognitive outcomes they have found benefit with slower rewarming rates and smaller temperature gradients. The current recommendations are to rewarm at no more than 0.5°C per minute if the arterial outlet temperature is above 30°C, using an arterial to venous temperature gradient across the oxygenator of no more than 4°C. When the temperature is <30°C a maximum gradient of 10°C should be used.^84^

### Treatment of neurocognitive disorders

Delirium treatment is complex and hampered by an incomplete understanding of the pathogenesis and heterogeneity of this complex disorder.^85^ Cardiac surgical patients require close monitoring, and the intensive care unit (ICU) environment exacerbates delirium. The first step must be to recognise the condition and structured, protocolised ICU care using validated tools such as e.g., the Confusion Assessment Method for the Intensive Care Unit (CAM-ICU) should be implemented.^86^ After a physical, physiological and biochemical assessment to exclude treatable causes of confusion and optimising pain control, non-pharmacological management should be standardised. This includes measures to help orientation, cognitive stimulation, consistent nursing staff and promotion of routine and a normal circadian rhythm. Evidence in general ICU patients suggests that dysfunctional cerebral autoregulation contributes to the development of delirium, but it is unclear whether personalised blood pressure targets will improve outcomes.^87^

Few pharmacological strategies have a convincing evidence base and some, for example benzodiazepines, are associated with increased delirium and a prolonged ICU stay.^88^ Once extubated, sedatives and antipsychotics should be reserved for patients posing a risk to themselves. Dexmedetomidine has been shown in a meta-analysis to reduce the incidence of delirium in postoperative cardiac patients.^89^ However the DECADE placebo controlled randomised trial recruited 798 cardiac surgical patients and found no reduction in delirium.^90^ In addition, the authors postulated that the hypotension induced by the study drug may have confounded the study by worsening delirium. Attempts to focus the use of dexmedetomidine upon subgroups that are of increased risk of delirium have involved perioperative study of older (greater than 65 years) patients undergoing non-cardiac surgery. Moderate-sized RCTs have produced conflicting results.^91,92^

### Time course and prognosis of neurocognitive disorders

Deficits in cognition resolve gradually, with most patients returning to baseline function within 12 months of surgery.^6^ The strongest predictors of recovery are greater educational attainment, less severe cognitive deficit, and functional status at 6 weeks after surgery.^93^ The existence of long-term adverse cognitive trajectories after major surgery is debated. Some studies that have looked at the persistence of cognitive deficits at up to 5 years postoperatively after CABG have shown an incidence of up to 42% at 5 years.^94^ If valid, this should be a factor in the consent process.

However, later studies using control groups have suggested that there may be a background level of cognitive decline due to cerebrovascular disease; in effect, a slow progression of a vascular dementia-like process. Several case–control studies comparing CABG patients with matched patients who did not have surgery show comparable rates of decline.^6^ Other authors have associated reduced cardiac output, irrespective of surgery, with cognitive decline.^95^

Long-term neurocognitive follow up is challenging and resource intensive. It is also vulnerable to confounding factors because patients who remain well will be more likely to complete the structured assessments required. A meta-analysis from 2005 of data comparing CABG with OPCAB concluded that variability across trials was too high to rule out clinically important differences in neurocognitive dysfunction.^96^

Perhaps the best available data comparing CABG and OPCAB, the CORONARY trial, enrolled 4752 patients and reported a Montreal Cognitive Assessment at 1 year in approximately one quarter of recruited patients; they were unable to report these data at all at 5 years.^97,98^ Patients undergoing OPCAB performed slightly better on one single test at discharge from hospital. Nevertheless, CORONARY patients showed no cognitive decline at 1 year and no difference between CABG and OPCAB at 30 days and 1 year. This may be the best attainable evidence that there is no significant long-term neurocognitive decline caused by the use of CPB.^98^

## Other neurological complications

### Seizures

Seizures complicate recovery after cardiac surgery in up to 1% of patients.^3^ However, this is likely to be an underestimate as some studies find the incidence of nonconvulsive seizures to be higher than 8% in critically ill patients. This may represent a treatable cause of poor neurologic outcomes but progress in this area will require more extensive use of EEG technology in the perioperative period. Gofton et al. used continuous EEG and found a 3% incidence of seizures, concluding that this level of monitoring was not cost-effective.^99^

The pathophysiology of seizures is multifactorial, involving cerebral ischaemia due to hypoperfusion or embolization, biochemical disturbances and pharmacologic agents. Although a majority of anaesthetic drugs have been implicated in seizure activity to some degree, the anti-fibrinolytic tranexamic acid when used at high doses (>80 mg/kg) is thought to be the main culprit.^100^ This is based upon retrospective analyses showing an increase of seizure incidence from ∼1% historically to approximately 7% after the introduction of high dose tranexamic acid.

Intrathecal and brain slice application of TXA in animal model systems can elicit seizure-like activity. TXA is thought to act as a competitive inhibitor of glycine receptors, reducing the levels of tonic inhibition and thus having a pro-convulsant effect at the network level. This effect was attenuated by the application of isoflurane and propofol to the mouse brain slices, and CSF levels lag behind serum levels, which may explain why seizures are often noted upon emergence from anaesthesia but not noted on intraoperative EEG. TXA is renally eliminated and clinical data suggest that patients with chronic renal failure are at higher risk for seizures.

### Coma

Coma is known to occur after CPB (Table 3), but the incidence will depend on the definition used, and upon institutional protocols for sedation and extubation after surgery.^101,102^ In a retrospective analysis, Rodriguez et al. describe 112 patients who had no consistent motor or verbal response after holding sedation for 24 h^4^ All but three had undergone on-pump surgery and this represented 2.8% of the records screened. Acute kidney injury, anaemia and emergency surgery were associated with a longer period of coma. In addition to these associations, many of the risk factors are common with those for seizures, stroke, delirium and postoperative cognitive dysfunction. Most authors consider these symptoms to lie along a broad continuum of neurologic dysfunction with overlapping clinical features.

**Table 3.** Causes of delayed emergence from anaesthesia and coma.

Cardiopulmonary bypass	Prolonged bypass time Prolonged circulatory arrest
Pharmacokinetic causes	Inappropriate dose (absolute or relative) Impaired transport/distribution Impaired metabolism (liver failure, hypothyroidism) Impaired elimination (renal failure) Duration of surgery Drug interaction
Pharmacodynamic causes	Genetic variations Hypothermia Drug interaction
Metabolic alterations	Hypoglycemia/hyperglycemia Hyponatremia/hypernatremia Acidosis
Rare causes	Hypoperfusion/ischemia Intracranial hemorrhage Thrombosis Seizure (including non-convulsive status epilepticus) Serotonin syndrome Central anticholinergic syndrome Myxoedema coma Hepatic encephalopathy Conversion disorder/functional coma

### Visual disturbance

Ophthalmologic complications after CPB range from subtle defects to complete visual loss. Intraoperative evidence has shown that transient microvascular occlusion occurs in most, if not all patients studied from an elective cohort, with diabetics and those with known cerebrovascular disease excluded.^103^ The majority of these occlusions resolve during the first postoperative week. Shaw et al. conducted a prospective study of CABG patients using pre- and postoperative clinical examination by a specialist neurologist and included a control group of vascular surgery patients who did not undergo CPB. This early and high-quality study showed that 13% of patients had pre-morbid ophthalmological abnormalities and 26% of patients developed new abnormalities in the perioperative period.^104^ These included retinal infarction (17%), retinal emboli, visual field defects, loss of visual acuity and the development of Horner’s syndrome. There were no new deficits in the control group; however, OPCAB patients would be the gold standard comparator to address the specific risk attributable to CPB.

Perioperative ischaemic optic neuropathy (ION) often presents with profound visual loss in one or both eyes. Kalyani et al. reported retrospective data from 9906 cases over 9 years and found a 0.11% risk of this devastating condition, which is poorly understood and has limited treatment options.^7^ Only 205 of these cardiac surgical cases were performed without CPB and, whilst none of these patients developed ION, this result could occur by chance alone. A history of non-coronary arterial disease increases risk substantially. The pathophysiology is opaque, but the focus has been upon determinants of oxygen delivery. In this scenario low flow, low pressure and haemodilution caused by CPB may explain the elevated rates of ION in cardiac surgery compared to other procedures.

A saccadic palsy has also been described, occurring most commonly after surgery on the aortic valve or ascending aorta.^8^ This has been associated with dysarthria, gait changes and emotional lability and as a syndrome resembles progressive supranuclear palsy. This is thought to be rare, but the combination of vague symptoms and subtle signs of upward gaze abnormality may mean that it is underdiagnosed. The pathophysiology probably involves ischaemic damage to midbrain nuclei and the procedures reported confer a high risk of other forms of perioperative stroke. However, in the majority of cases there were no focal lesions upon MRI of the brain.

### Peripheral nerve lesions

Peripheral nerve dysfunction due to operative positioning is described after a variety of surgical procedures, however cardiac surgery patients are at elevated risk.^105^ Rarer are entities such as brachial plexopathies, probably related to sternal retraction.^106^ The four cases of Horner’s syndrome reported by Shaw et al. were all accompanied by clinical evidence of brachial plexus damage.^104^ However, these are attributable to the specific surgical technique and patient positioning, rather than the use of CPB alone. The exception to this rule may be phrenic nerve injury, which occurs in up to 26% of patients when investigated by chest radiography, diaphragmatic ultrasound and phrenic nerve conduction studies.^9^ Three quarters of the injuries described by DeVita and colleagues in this study were left sided. This nerve is most vulnerable anatomically to hypothermic injury caused by packing the arrested heart in ice. Alternatively, damage may occur during the dissection to expose the internal mammary artery. Unilateral phrenic nerve palsy tends to be relatively well tolerated and to recover within 3–6 months. Bilateral phrenic nerve damage, although rare, can be a difficult to diagnose cause of slow weaning from mechanical ventilation. In patients with pre-existing respiratory pathology it may be prudent to avoid topical cooling of the heart, to use a cardiac insulation pad or to monitor phrenic nerve function intraoperatively.^107^

### Chorea

A chorea-related motor disorder has been described after CPB, particularly in series of paediatric patients and a single series of adult patients undergoing pulmonary endarterectomy (PEA).^10,108^ This has been termed “post-pump chorea”. In the paediatric literature the affected patients spent a longer time on bypass, were cooled to a lower temperature, and were more likely to have had DHCA. PEA patients with chorea were younger and had been re-warmed more rapidly than controls. Taken together with the lack of focal cerebral lesions on cross-sectional imaging, the authors suggest that this phenomenon is related to temperature change or the extended use of CPB. However, as with the saccadic disorders it is difficult to establish causation. In some patients the chorea persisted and none of the paediatric patients followed a normal developmental course following surgery.^10^

## Conclusions

Huge progress has been made in the safe perioperative management of CPB. This has been used to extend the benefits of cardiac surgery to higher risk patients in terms of age, comorbidities and procedural risk factors. The advent of minimally invasive techniques and off-pump surgery mean that patients presenting for conventional bypass and DHCA may also be a higher risk subgroup. However, it should not be assumed that neurological complications described here are attributable to CPB alone. In principle, any aspect of the individual patient, the disease process and the perioperative management should be examined and optimised. The emerging consensus is that, in experienced hands, CPB is equally as safe from a neurological point of view as alternatives that exist.

Nonetheless, neurological complications are common and must be anticipated, particularly with valve procedures, longer procedures and any surgery involving DHCA. Most strokes are clinically evident within 48 h and should be managed by a specialist multidisciplinary team. Delirium is likely to be under-recognised and the risk may be in excess of 50% in older patients. Long term adverse cognitive trajectories, whatever the cause, will remain a clinical challenge. Taken together, there is huge scope for development in perioperative care but also the potential to create significant dependence, even if the cardiac procedure is a technical success. Capturing this fragile balance and sharing it with patients is central to informed consent.
